# Supplementary material for: Comparison of haemoglobin concentration measurements using HemoCue-301 and Sysmex XN-Series 1500: A survey among anaemic Gambian infants aged 6–12 months
Source: PLoS One. 2024 Nov 25;19(11):e0310577. doi: 10.1371/journal.pone.0310577 (PMC11588255; doi:10.1371/journal.pone.0310577)
Supplement: S1 File — (PDF) [file pone.0310577.s001.pdf]

S1 File. Raw dataset used in this analysis

ID	HemoCue-301	Sysmex XN-1500	sex	Age, months
0003H	9.6	9.7	Male	8.7
0004F	9.9	9.9	Female	9.2
0008E	10.2	10.5	Male	7.9
0010B	10.7	10.3	Female	10.7
0011H	10.8	11.3	Male	7
0012F	9.6	10.2	Female	9.4
0017E	10.5	10.9	Male	11.2
0020H	9.9	8.9	Male	6.4
0021A	9	7.5	Male	7.2
0022K	10.4	10.2	Male	6.1
0023C	10.4	10.5	Female	6.2
0024B	9.9	7.5	Female	7.5
0026E	10.2	10.5	Female	7.2
0028G	10.1	9.4	Female	8.3
0029D	7.8	7.3	Female	6.8
0030J	10.2	9.9	Female	6.3
0031K	10.2	9.7	Male	6.4
0037A	8.6	9.5	Male	6
0042H	10.7	10.6	Female	8
0043E	10.1	9	Female	7.1
0045A	9.8	9.6	Female	7
0049B	10	9.1	Male	9.7
0051F	10	7.2	Female	8.6
0052J	10.5	10	Male	9.9
0058K	10	11.2	Female	6.4
0059A	10.1	10.9	Female	6
0063J	10.9	10	Female	6.4
0064G	10.9	10	Female	8.8
0065B	10.5	9	Male	7.5
0066H	10.4	9.4	Female	10.2
0070F	10.3	9.7	Male	6.8
0071J	10	9.7	Female	10.4
0073K	10.6	10.5	Female	6.9
0074H	7.8	9.3	Male	6.6
0081B	10.2	8.7	Female	10
0085E	10.1	9.2	Female	10.1
0089J	10.3	9.8	Female	9
0090E	9.5	8.6	Male	8
0095H	9.7	10.1	Female	8.3
0097D	10.6	10.4	Female	11.1
0098F	8.3	9.8	Female	10.1
0101A	10.4	9.4	Male	11.1
0103C	9.3	10.2	Female	9.6
0109D	8.6	8	Male	6.2
0110J	10.5	9.7	Male	7.4
0112E	10.7	11.2	Male	6.3

0121G	10	10.9	Female	6.4
0131D	10.4	9.8	Female	6.8
0135K	9.5	8.9	Male	7.7
0146G	9.1	8.2	Male	6.4
0148K	10.7	10.3	Male	6.2
0151E	10.1	11.2	Male	6.7
0154G	10.3	10.4	Male	7.4
0159F	10.5	9.8	Male	6.1
0160B	9.3	9.4	Male	6.5
0164K	10.2	9.4	Male	7.6
0165J	10.8	9.7	Female	11.3
0169G	9.2	7.9	Male	6.8
0176F	10.1	9.7	Female	11.1
0179J	10.7	10.1	Male	6.3
0180E	9.7	9.1	Male	6.1
0181C	10.8	10.9	Female	6.4
0186B	8.6	8.4	Male	10.8
0188F	10.3	8.9	Female	6.1
0189K	9.1	10.4	Male	6.9
0191J	10.8	9	Male	5.3
0192G	10.8	10.3	Female	7.1
0193K	10	9.9	Female	6.8
0194H	10.6	10.5	Female	6
0196A	9.5	9.8	Female	11.2
0201K	10.6	10	Male	6.4
0210A	10.1	10.1	Male	6.8
0212B	10.5	9.9	Male	6.3
0213H	9.3	8.8	Male	6.2
0221F	9.7	9.6	Female	6.3
0223B	10.8	10.2	Male	6.4
0224E	10.9	10.4	Male	8.9
0228K	10.9	9	Female	6.2
0229A	10.7	11.1	Female	6.3
0232A	10.2	9.1	Male	6
0234J	10.8	11.2	Female	6.3
0235H	10.3	9.6	Female	6.1
0236B	10.2	9.1	Female	7.1
0237D	10.9	8.9	Female	6.8
0238F	10.6	8.9	Female	6.5
0239K	10.6	9.4	Female	6.4
0240H	10.1	9.8	Male	10.5
0243C	8.8	7.6	Male	6.5
0254C	10.9	11.8	Female	7.6
0255A	10.2	7.9	Female	7.6
0257F	10.6	10.7	Male	6.7
0260G	10.1	9.1	Female	7.5
0265E	10.2	8.5	Male	7.1
0272D	10.9	11.1	Male	6.6
0273J	9.9	10.9	Female	6.2
0277C	10.5	10.3	Female	7.3

0278A	10.9	10.6	Female	6.5
0280F	10.7	10.7	Male	6.4
0290B	9.5	9.7	Male	6.4
0294K	10.9	9.6	Male	8.2
0298C	9.8	10.1	Female	10.4
0299G	9.4	10.5	Male	6.2
0300K	10.5	10.5	Female	7.2
0301E	10.8	10.4	Female	7.3
0316G	10.8	9.8	Female	6.2
0317H	10.2	8.9	Male	6.5
0318K	10.5	9.5	Male	6
0320G	10.3	11.2	Female	6.2
0327K	9.2	8.8	Male	9.1
0328H	10.8	10.9	Male	6.5
0330D	10.1	8.5	Female	11.3
0331G	10.8	11.1	Female	9.8
0334C	10.2	10.1	Female	6.5
0342F	10.5	10.3	Female	6.8
0343A	10.7	10.3	Female	6.1
0344K	10.4	12.3	Female	6.4
0347E	9.1	9.4	Female	8.1
0349G	10.1	11.4	Female	8.1
0351J	9	7.7	Male	6.8
0352G	10.8	10.8	Female	6.1
0353K	8.7	9.7	Male	6.3
0359E	10.3	10.5	Male	9.1
0361C	10.3	9.1	Female	7
0362A	10.5	10.1	Male	6
0363G	10.7	10.2	Female	9.8
0364J	10.5	9.4	Male	6.3
0365H	10.8	9.9	Male	6.2
0370A	9.9	10.5	Female	6.8
0374F	8	7	Male	10.8
0375K	10.9	11.4	Male	9.6
0376J	10.7	10.6	Male	6.3
0377G	10.5	9.5	Female	9.3
0380H	10	9.2	Male	6.1
0381A	9.6	9.6	Male	6.3
0386E	10.1	10.1	Male	6.2
0388G	10.1	9.7	Male	6.2
0390J	9.7	9.9	Male	6.1
0393F	9.5	10.4	Male	9
0397A	10	9.3	Female	6
0401H	10.7	10.5	Female	6.6
0402F	10.6	9.9	Male	6.6
0404K	10.3	9.1	Male	9.5
0406D	10.6	10	Male	6.4
0407E	10	11.6	Female	10.2
0414H	9.8	9.7	Female	6.6
0416A	9.4	8	Female	8.5

0417B	9.6	9.5	Female	8.6
0418D	8.6	7.9	Female	8
0419E	10.1	10.6	Male	6.1
0420J	10.6	9.9	Female	6.4
0423F	9.4	9.5	Male	9.2
0424D	10.1	10.4	Female	6.3
0426C	10.7	10.8	Male	6.2
0430G	8.7	8.8	Male	6.1
0431B	10.8	10.1	Female	7.3
0433D	10.3	9.9	Male	6.7
0436F	9.9	10.2	Male	6.5
0437K	10.1	10.6	Female	6.4
0438H	9.3	10.1	Male	10.3
0439J	10.8	10.6	Male	6.7
0440E	10.7	10.7	Male	6.2
0441C	10.1	9.2	Male	6.1
0442A	9.1	10.1	Female	6.1
0444J	10	9.5	Male	8
0445H	10.5	9.4	Male	6.1
0446B	10.4	9.1	Female	6.7
0448F	9.8	9.2	Male	6.2
0449K	10.8	10.3	Female	6.3
0453H	9.7	9	Male	8.1
0454F	10.8	10.8	Male	7.8
0455K	10.8	9.9	Female	9.2
0456J	10.3	9.4	Male	7.2
0458E	9.2	8.7	Female	6.3
0461F	9.8	9.5	Female	6
0464E	9.1	10.5	Male	7.2
0466G	10.9	9.1	Male	7.9
0467H	9.4	6.7	Male	7.3
0468K	10.3	9.5	Male	6
0469A	10.7	10.1	Female	11.2
0470D	10.4	9.7	Male	6.1
0472H	10.8	10	Female	7
0473E	10.1	9.5	Female	6.2
0478J	9.9	10.9	Female	6.3
0479B	10.4	8.6	Female	6
0489F	10.3	9.6	Female	10.1
0490H	9.9	9.7	Male	6.1
0491A	10.1	9.2	Female	9.5
0493C	10.3	9.7	Male	7.4
0494B	10.8	9.5	Male	6.2
0495F	10.9	10.1	Female	8.5
0500E	9.7	8.6	Female	7.6
0501C	10.8	9.8	Male	6.5
0504J	10.7	10.1	Male	9.6
0506B	10.5	8.6	Female	6.1
0508F	8.6	7.1	Female	6.2
0509K	8.2	7.2	Male	11.5

0513E	8.6	8.8	Male	10.9
0514C	8.9	7.7	Male	12
0517F	10	10.4	Male	9.6
0518J	10.9	9.8	Male	11.8
0519B	10.9	9.9	Female	6.1
0521E	9.1	11.4	Female	6
0523J	9.7	9	Female	10.4
0528A	10.8	13.5	Male	9.6
0529F	10.7	9.5	Male	6.4
0538G	10.9	10.8	Female	6.6
0543D	10.8	8.8	Male	6
0547K	8.1	8.1	Female	8.6
0552F	10.9	9.9	Female	8.8
0553A	10.6	9.3	Female	8.7
0554K	10.9	10.7	Female	6.9
0555J	10	10.3	Female	7.4
0556D	10.8	10.1	Female	8.1
0559G	10.1	9.6	Female	6.9
0560F	8.9	9.5	Male	7.7
0561J	10.6	10.7	Male	6.6
0568D	10.5	9.4	Female	9.4
0570J	10.4	10	Female	6.3
0574D	9.6	10.1	Female	7.5
0582J	8.7	8.9	Female	8.2
0583B	9.6	9.4	Female	7.5
0584E	10	10.3	Female	6.1
0587H	9.2	10	Female	7
0592B	10.6	11.9	Male	6.8
0593H	9.9	9.2	Female	6.5
0596J	10.6	8.7	Female	6.2
0600C	10.7	10.2	Male	6.1
