# Supplementary material for: Comparison of haemoglobin concentration measurements using HemoCue-301 and Sysmex XN-Series 1500: A survey among anaemic Gambian infants aged 6–12 months
Source: PLoS One. 2024 Nov 25;19(11):e0310577. doi: 10.1371/journal.pone.0310577 (PMC11588255; doi:10.1371/journal.pone.0310577)
Supplement: Attachment — Submitted filename: Review PNTD_D_24-33800.docx [file pone.0310577.s002.docx]

## Review of the manuscript: “Comparison of haemoglobin concentration measurements using HemoCue-301 and Sysmex XN-Series 1500: a survey among anaemic Gambian infants aged 6-12 months” (PNTD-D-24-33800)

### Summary

This article evaluated the accuracy of the point-of-care Hb measurement assay HemoCue-301 in measuring Hb levels in anaemic children against the reference assay Sysmex XN-1500. The absolute and proportional differences between measurements from the two assays were analysed with the Passing-Bablok regression. This article found that larger bias/differences were present in lower Hb levels, that were outside of the acceptable difference of ±7.0%.

### Comments

- Methods
  - Please include definitions (Hb level ranges) of severe, moderate, mild anaemia
  - Kindly describe the definitions of Absolute Bias and Proportional Bias used in this study (the equations in the caption of Figure 1) in this section, so the readers know where the number in the Results section came from
  - Lines 132-133: “We defined absolute and proportional bias when the confidence intervals excluded 0 and 1 for the intercept or the slope respectively.” I am a bit unsure of what this sentence means; perhaps reword it to clarify?
- Results
  - Table 1 and Figure 1 were not referred to in the text
  - Participant demography: how many participants have severe, moderate, mild, and no anaemia? Also consider describing age, sex, and other factors that may impact Hb levels among participants
  - Lines 143-144: “One participant was classified as severely anaemic (haemoglobin <7.0 g/dl) …” In line 149 severe anaemia was defined as 6.5 g/dL, this is contradictory
  - Lines 148-151: “For severe anaemia (haemoglobin concentration at 6.5 g/dL), Hemocue measurements showed an absolute bias of 1.72 g/dL (95% CI: 1.37 to 2.10 g/dL) and a proportional bias of 26.5% (95% CI: 21.1% to 32.3%).” Is the listed absolute and proportional bias applicable to 6.5 g/dL or to the entire range of severe anaemia (anything below 6.5 G/dL)?
  - Lines 162-163: “the magnitude of bias is inversely proportional to haemoglobin concentration” Based on Figure 1, the bias was also increasing but to the negatives the higher the Hb concentration
  - Lines 170-174: This is a repeat of what has been written in the Results section, consider removing
  - Lines 174-175: “… while those with a haemoglobin concentration of 11.0 g/dL showed marginal or non-existent bias.” What made you say this? What was the limit of being "marginal"? At 11 g/dL according to Table 1, the proportional bias was -7.3%, which was outside of the acceptable limit of ±7.0%.
  - Lines 175-176: “Similarly, a study conducted in Laotian children reported a higher bias in anaemic compared to non-anaemic children (22).” Only anaemic children were enrolled for this study, how is this comparable to the Laotian study?
  - Lines 199-215: Consider putting these in the Introduction/Background section instead
- Conclusion
  - Lines 242-243: “Our study indicated that bias due to HemoCue-301 depends on the actual Hb concentration, and seems unacceptably high at Hb values below 8.0 g/dL.” At 8.0 g/dL the proportional bias was 14.0%, which was also beyond the acceptable value of ±7.0%. Consider calculating a range of Hb levels where the proportional bias is acceptable (±7.0%) instead.
